# Nanosuspensions and Microneedles Roller as a Combined Approach to Enhance Diclofenac Topical Bioavailability

**DOI:** 10.3390/pharmaceutics12121140

**Published:** 2020-11-25

**Authors:** Rosa Pireddu, Michele Schlich, Salvatore Marceddu, Donatella Valenti, Elena Pini, Anna Maria Fadda, Francesco Lai, Chiara Sinico

**Affiliations:** 1 Dipartimento di Scienze della Vita e dell’Ambiente, Sezione di Scienze del Farmaco, CNBS, Università degli Studi di Cagliari, 09124 Cagliari, Italy; rosapireddu25@gmail.com (R.P.); michele.schlich@unica.it (M.S.); valenti@unica.it (D.V.); mfadda@unica.it (A.M.F.); sinico@unica.it (C.S.); 2 Laboratory of Nanotechnology for Precision Medicine, Istituto Italiano di Tecnologia, 16163 Genova, Italy; 3 Istituto di Scienze delle Produzioni Alimentari (ISPA)-CNR, Sez. di Sassari, 07040 Baldinca, Italy; salvatore.marceddu@cnr.it; 4 DISFARM, Sezione di Chimica Generale e Organica “A. Marchesini”, Università degli Studi di Milano, Via Venezian 21, 20133 Milano, Italy; elena.pini@unimi.it

**Keywords:** nanocrystals, microneedles, nanosuspension, dermal delivery, diclofenac

## Abstract

Topical application of the anti-inflammatory drug diclofenac (DCF) reduces the severity of systemic unwanted effects compared to its oral administration. A number of transdermal formulations are available on the market and routinely used in clinical and home-care settings. However, the amount of DCF delivered across the skin remains limited and often insufficient, thus making the oral route still necessary for achieving sufficient drug concentration at the inflamed site. In attempting to improve the transdermal penetration, we explored the combined use of DCF nanosuspensions with a microneedle roller. Firstly, DCF nanosuspensions were prepared by a top-down media milling method and characterized by spectroscopic, thermal and electron microscopy analyses. Secondly, the pore-forming action of microneedle rollers on skin specimens (ex vivo) was described by imaging at different scales. Finally, DCF nanosuspensions were applied on newborn pig skin (in vitro) in combination with microneedles roller treatment, assessing the DCF penetration and distribution in the different skin layers. The relative contribution of microneedle length, nanosuspension stabilizer and application sequence could be identified by systemically varying these parameters.

## 1. Introduction

Diclofenac, 2-[(2,6-dichlorophenyl)amino]phenylacetic acid (DCF), is one of the most powerful and commercially successful non-steroidal anti-inflammatory drugs (NSAID). Similar to other drugs from this class, DCF is associated with dose-dependent gastrointestinal, cardiovascular and renal toxicity. Since its advent on the market in 1973, a number of strategies based on pharmaceutical technology were explored to improve the efficacy of DCF and to limit the severity of its adverse effects [1]. For instance, moving from the oral route to a topical cutaneous administration allows to effectively treat a number of localized inflammatory conditions (e.g., musculoskeletal, epidermal) while reducing the amount of DCF absorbed systemically and the consequent organ toxicities [2,3]. This is of utmost importance for the treatment of chronic inflammatory diseases, where a continuous systemic exposure to the drug might result in unacceptable side effects [4]. DCF is characterized by low water solubility in its free-acid form, as its carboxylic moiety is involved in the formation of a hydrogen bonds-stabilized dimer and therefore not available to interact with the solvent. The low solubility severely limits the bioavailability of DCF, which is therefore usually included in medicinal products as a sodium, potassium or organic salt of the parent compound [5]. However, the higher solubility of DCF salts comes at the expense of lipophilicity, which will be significantly reduced compared to the free acid form. This directly affects the skin permeability of DCF, which was shown to be reduced by up to 3 orders of magnitude when the sodium salt was employed [6]. Thus, alternative strategies aimed at increasing DCF water solubility, while maintaining the lipophilic character, could improve the topical delivery of the drug.

The nanocrystal technology represents one of such approaches. Nanocrystals can be defined as nanoparticles of pure drug with an average diameter below 1 µm, suspended in a water and/or water-miscible solvent and stabilized by surfactant(s) or polymer(s) [7]. The nanosizing process enhances drug skin penetration due to three combined effects: (1) It increases drug saturation solubility and, consequently, the concentration gradient between the formulation and the skin surface; (2) it boosts dissolution rate due to the larger particle surface area; (3) it promotes the adhesion of nanocrystals to the skin [8]. DCF nanocrystal suspensions (NS) previously developed in our lab showed enhanced skin accumulation compared to coarse suspensions and proved to be efficient in reducing skin inflammation in vivo [9,10]. Recently, in the attempt of further improving the efficacy of this technology, DCF NS were coupled with a well-known skin penetration enhancer (Transcutol P^®^, Gattefossé) [11]. Disappointingly, the presence of Transcutol influenced the formation of nanocrystals and reduced the amount of DCF permeated in the skin in vitro. Another approach for disrupting the barrier properties of the skin and allowing more drug in the underlining tissues relies on the use of microneedles. Microneedles have been introduced to increase skin permeability by creating micron-sized pores across the stratum corneum [12]. In the last decade, great effort has been devoted to the development of different microneedle technologies (solid, coated, hollow, dissolvable) to deliver small- and macro-molecules, vaccines and nanoparticles across the skin barrier [13,14,15]. Microneedles are often embedded in a patch for convenient application and retention in a specific site. When larger skin areas need to be treated, a microneedle roller could prove useful [16]. A microneedle roller is a medical device composed by stainless steel or polymeric microneedles mounted on a cylindrical surface that can be rolled on the skin, creating a periodic pattern of micropores. Microneedles with different needle lengths, from 20 to 2000 micron, are commercially available and used both for cosmetic and therapeutic purposes [17]. The combination of nanotechnology with advanced, minimally invasive devices -such as microneedles or needle-free jet injectors- might further improve the local delivery of therapeutics [18,19]. In the present work, the combination of nanocrystal technology with the use of a microneedle roller was investigated as a potential method to enhance DCF delivery to the skin. DCF NS prepared by a top-down method were characterized and topically applied to microneedle-treated porcine skin in vitro. For a better comprehension of the potential of such combination, three different microneedle lengths, two DCF NS formulations and two application schemes (microneedling before or after the topical application of DCF NS) were investigated.

## 2. Materials and Methods

### 2.1. Materials

**Figure 1:**
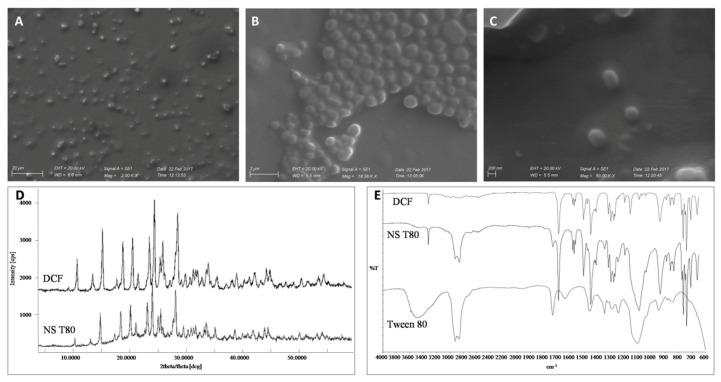
Characterization of NS T80 diclofenac (DCF) nanosuspensions. (**A**–**C**) Scanning electron microscopy images, scalebars 20 µm, 2 µm and 200 nm, respectively. (**D**) X-ray powder diffraction of bulk DCF and DCF nanosuspensions. (**E**) Fourier transform infrared spectroscopy of bulk DCF, DCF nanosuspensions and Tween 80.

Diclofenac sodium salt, Polysorbate 80 (Tween 80) and Poloxamer 188 (P188) were purchased from Galeno (Comeana, Italy). Stainless steel microneedle rollers (Derma-Q, 0.2 mm, 0.5 mm and 1.5 mm) were obtained from DONGBANG Acupuncture Inc. (Boryeong, Korea). Three different models of Derma-Q were used: Q11-needle length 0.2 mm, Q12-needle length 0.5 mm and Q14-needle length 1.5 mm. All types of Derma-Q possess 24 circular arrays of 8 needles each (total 192 needles) in a cylindrical assembly (Figure 1). All the other reagents were purchased from Sigma–Aldrich (Milan, Italy).

### 2.2. Preparation of DCF Acid

Diclofenac acid crystal form was obtained following the procedure reported in a previous work [20]. Briefly, a saturated aqueous solution of diclofenac sodium salt was acidified with diluted HCl until a white precipitate of DCF acid was observed. The precipitate was filtered out, washed with bidistilled water to remove residual HCl and dried at 40 °C overnight.

### 2.3. Preparation and Characterization of DCF Nanosuspensions

Diclofenac nanosuspensions were prepared using a wet media milling technique. The bulk drug (1% *w*/*w*) was dispersed in an aqueous solution of Tween 80 or P188 (0.5% *w*/*w*) using an Ultra Turrax T25 basic for 5 min at 6500 rpm (Table 1). This coarse suspension was divided in 1.5 mL conical microtubes containing about 0.4 g of 0.1–0.2 mm yttrium-stabilized zirconia-silica beads (Silibeads^®^ Typ ZY Sigmund Lindner, Warmensteinach, Germany). The microtubes were oscillated at 3000 rpm for 60 min using a beads-milling cell disruptor equipment (Disruptor Genie^®^, Scientific Industries, Bohemia, NY, USA). The obtained nanosuspensions of each microtubes were gathered and then separated from the milling beads by sieving. Drug/surfactant ratio (*w*/*w*) was 2:1. The average diameter and polydispersity index (PI; a measure of the size distribution width) of the nanosuspensions were determined by photon correlation spectroscopy (PCS) using a Zetasizer nano (Malvern Instruments, Worcestershire, UK). Zeta potential was measured using the Zetasizer nano by means of the M3-PALS (Phase Analysis Light Scattering) technique. Just before the analysis, the DCF nanosuspensions were diluted with bi-distilled water. A long-term stability study of DCF nanosuspension stored at 25 ± 1 °C was performed by monitoring average size, polydispersity and surface charge for 90 days.

### 2.4. Solid Phase Analysis

#### 2.4.1. Differential Scanning Calorimetry

The DSC curves of the different samples were recorded on a Perkin Elmer DSC 6 (Perkin Elmer, Waltham, MA, USA). differential scanning calorimeter calibrated with indium. The thermal behavior was studied by heating 2.5 mg samples in aluminum crimped pans under nitrogen gas flow. The samples were heated from 50 °C to 350 °C at heating rates of 10 °C/min.

#### 2.4.2. Fourier Transform Infrared Spectroscopy

Infrared absorption spectra were recorded on a Perkin Elmer FTIR Spectrometer (Perkin Elmer, Waltham, MA, USA). “Spectrum One” in the middle infrared region (4000 and 650 cm^−1^). The liquid samples were analyzed as such on NaCl disks while for solid samples an ATR (Attenuated Total Reflection) Sampling Accessory consisting of diamond crystal was used (Perkin Elmer, Waltham, MA, USA). Analyses were performed with 32 signal-averaged scans collected at a spectral resolution of 4 cm^−1^.

#### 2.4.3. X-Ray Powder Diffractometry

The XRPD analyses were carried out by using a Rigaku MiniFlex diffractometerv (Rigaku Corporation, Tokyo, Japan). The setting parameters were a CuKα radiation detector (λ = 1.54056 Å), a voltage of 30 kV, current of 15 mA, a scan angular speed of 2°/min, and a scan step time of 2.00 s in the range from 3° to 60°. The results were then obtained as peak height (intensity) versus 2*θ*.

### 2.5. Solubility Studies

The water solubility of DCF was measured for the DCF coarse suspensions and nanosuspensions. The formulations (*n* = 3) were kept under constant stirring in a thermostated bath at 37 °C. After 72 h, samples were withdrawn and centrifuged at 13,000 rpm for 60 min; the supernatant was centrifuged again at 13,000 rpm for 30 min. Then, 0.2 mL of the supernatant was diluted with methanol and analyzed by HPLC for DCF content.

### 2.6. Diclofenac Acid HPLC Quantification

DCF content was quantified at 280.4 nm using a chromatograph Alliance 2690 (Waters, Milan, Italy), equipped with a photodiode array detector and a computer integrating apparatus (Empower 3). The column was a SunFire C18 (3.5 µm, 4.6 mm × 100 mm, Waters, Milan, Italy), and the mobile phase was a mixture of 30% water and 70% acetonitrile (*v*/*v*), delivered at a flow rate of 0.7 mL/min. A standard calibration curve was built up by using working standard solutions. Calibration graphs were plotted according to the linear regression analysis, which gave a correlation coefficient value (R^2^) of 0.999.

### 2.7. In Vitro Skin Penetration and Permeation Studies

Experiments were performed on newborn pig skin. One day old Goland Pietrain hybrid pigs (~1.2 kg) died of natural causes were provided by a local slaughterhouse. The skin was cut out surgically, stored at −80 °C, and pre-equilibrated in saline solution (0.9% *w*/*v* NaCl) at 25 °C, 24 h before the experiments. Penetration and permeation studies were performed non-occlusively using vertical Franz cells with a diffusion area of 0.785 cm^2^. Pig skin specimens (*n* = 6) were placed between donor and receptor compartments of the Franz cells, with the stratum corneum (SC) side facing the donor compartment.

**Figure 2:**
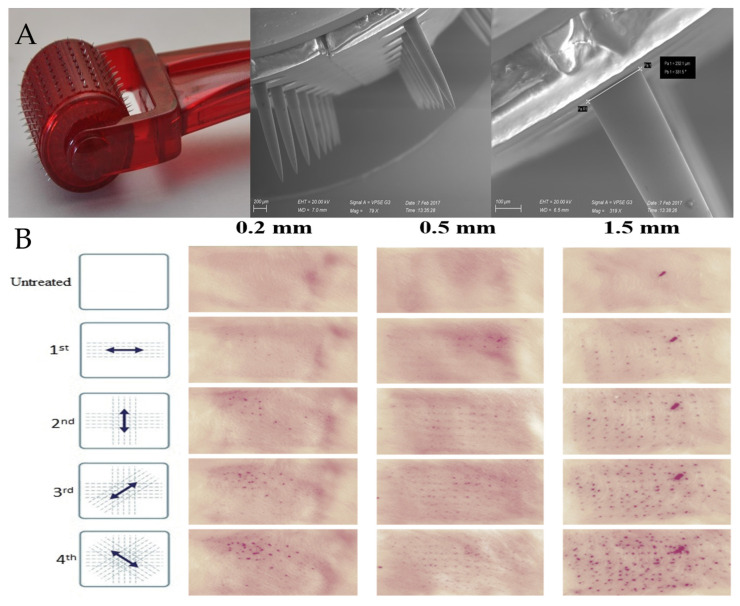
(**A**) Representative images (digital camera and SEM) of a stainless-steel microneedle roller, DermaQ^®^ (density of 192 microneedles and needle length of 1.5 mm). (**B**) Visualization of micropores on the skin after microneedle rolling in different directions. Pores were generated by the DermaQ^®^ with needle lengths of 0.2, 0.5 and 1.5 mm and visualized by the application of Nile Red nanosuspension.

Before the application of 100 µL nanosuspension over the skin surface, the skin specimens were perforated in four directions at 45 degrees from each other (Figure 2) with the DERMA-Q (Roll and Patch method). Alternatively, the skin samples were perforated in four directions directly after the application of 100 µL of nanosuspension (Patch and Roll method). DERMA-Q with needle lengths of 0.2, 0.5 and 1.5 mm were used. The microneedling was performed always by the same operator, and the formation of uniform pore patterns on the skin was assessed on random skin specimens by microscopy. As reference, 100 µL nanosuspension was applied over the skin surface, without application of microneedles roller.

The receptor compartment was filled with 6.5 mL saline solution, which was continuously stirred with a magnetic bar and thermostated at 37 °C to reach the physiological skin temperature (i.e., 32 °C). At regular intervals, up to 8 h, the receiving solution was entirely withdrawn, replaced with pre-thermostated (37 °C) fresh saline to ensure sink conditions and analyzed by HPLC for drug content. After 8 h, the skin surface was gently washed (3 times) with 1 mL of distilled water and then dried with filter paper. The stratum corneum (SC) was removed by stripping with adhesive tape Tesa^®^AG (Hamburg, Germany). Each piece of the adhesive tape was firmly pressed on the skin surface and rapidly pulled off with one fluent stroke. Epidermis was separated from dermis with a surgical scalpel. Tape strips, epidermis and dermis were cut, placed each in a flask with methanol and sonicated for 4 min in an ice bath to extract the drug. The tapes and tissue suspensions were filtered out and assayed for drug content by HPLC.

### 2.8. Visualization of Skin Pores Patterns

For visualization of the pores/microchannels produced by the DermaQ treatment, porcine skin samples were perforated as described in Section 2.7 and then stained for 3 min with a strongly colored nanocrystals suspension prepared with a water-insoluble fluorescent dye, Nile Red [21]. Following treatment, skin samples were immediately rinsed with saline to remove excessive dye. As the control, untreated porcine skin samples were similarly stained with Nile Red nanosuspension for 3 min and immediately rinsed with saline. Pictures of the treated and untreated skin samples were collected using a digital camera (Canon 500D, Tokyo, Japan).

### 2.9. Scanning Electron Microscopy

The morphology of nanosuspensions was evaluated using scanning electron microscopy (SEM) (Zeiss ESEM EVO LS 10, Oberkochen, Germany). Samples were placed on aluminum stub and dried under vacuum for 12 h, coated with gold and observed under an acceleration voltage of 20 kV in high vacuum modality with secondary electron detector.

Furthermore, SEM was employed to visualize and characterize the pores generated by the DermaQ treatment and to qualitatively investigate the distribution of nanocrystals over the skin surface and in the microchannels. Porcine skin specimens were placed onto filter paper soaked with PBS and kept in place with needles. Nanosuspension was then deposed on the skin with or without DermaQ microneedles pre-treatment (Roll and Patch).

SEM analysis of skin samples was conducted in two different ways: in high vacuum (HV) or in variable pressure (VP) or low vacuum mode. The skin samples were prepared for HV analysis by fixation in 2.5% glutaraldeyde in phosphate buffer 0.1 M-pH 7.2 overnight, and freeze-dryed by Edwards freeze drier apparatus (Edwards Vacuum, Burgess Hill, UK), mount onto aluminum stubs with conductive silver paint and Au-coating in an Edwards S150A Sputter Coater unit (Edwards Vacuum, Burgess Hill, UK). Skin specimens were then analyzed by SEM using an acceleration voltage of 20 kV in HV modality with secondary electron detector and backscattered electron detector (BSD). For analysis in VP mode, skin samples were mounted onto aluminum stubs and imaged without any pre-treatment, operating at 20 kV in VP. VP mode allows the analysis of biological samples in the hydrated state. Chamber pressure was regulated to 100 Pa and the images were taken with the VP secondary electron detector (VPSE) and backscattered electrons detector (BSD).

### 2.10. Statistical Analysis of Data

Results are expressed as the mean ± standard deviation. Multiple comparisons of means (one-way ANOVA with post-hoc Tukey HSD test) were used to substantiate statistical differences between groups, while Student’s t-test was used to compare two samples. Data analysis was carried out with the software package XLStatistic for Excel (Microsoft, Redmond, WA, USA). Significance was tested at the 0.05 level of probability (*p*).

## 3. Results and Discussion

The aim of this study was to verify whether the combined use of microneedles and nanosuspension technology could improve the DCF skin penetration and accumulation.

As shown in Table 1, the nanosuspensions used for this study were prepared using 1% Diclofenac acid and non-ionic surfactants, Tween 80 or Poloxamer 188, as stabilizer. For the development of dermatological products, non-ionic surfactants are preferred over ionic surfactants because of their better skin tolerability [22]. Moreover, both Tween 80 and Poloxamer 188 are able to efficiently stabilize the drug nanocrystals formed during the media milling process, limiting the crystal growth to the sub-micron range [10,23].

The nanosuspensions were prepared employing the wet media-milling technique, a robust and tunable process previously employed and optimized in our group for the production of DCF nanocrystals stabilized by Poloxamer 188 (NS P188) [10]. In addition to the NS P188, whose characterization has been previously carried out and published, here we prepared and characterized DCF nanosuspensions stabilized by Tween 80 (NS T80) for the first time.

Table 1 reports the PCS particles size, polydispersity index (PI) and Zeta Potential values of DCF nanosuspensions determined on the day of preparation (Day 0). The nanocrystals obtained after the milling process show a particle size ranging between 260 and 280 nm and are homogeneously dispersed (PI = 0.17). The difference in the average diameter between NS P188 and NS T80 is not statistically significant. The particle size of NS T80 was qualitatively confirmed by SEM (Figure 1A–C). The nanocrystals of both formulations showed a strongly negative Zeta Potential (>−35 mV). XRPD and ATR-FTIR studies were performed on the dried nanosuspensions in order to investigate changes in the crystalline state of DCF during the preparation. The results were then compared to those obtained for bulk DCF acid and Tween 80.

DCF acid is known to have two monoclinic polymorphs: HD1 and HD2 [24]. The crystal structure of DCF acid synthesized for this study was characterized by DSC, XRPD and ATR-FTIR, and it was in agreement with the description of the HD2 polymorph [9].

The DSC analysis of DCF acid exhibited a sharp endothermic peak (Tonset) centred at 177.14 °C, followed by sample decomposition, while NS T80 showed the melting endotherm centered at 158.3 °C. The melting point depression and the broadening of its endothermic peak detected for the nanocrystal formulation can be attributed to the presence of the stabilizer, as it was previously observed for NS P188 [9] (Figure S1).

The DCF acid ATR spectrum evidenced the characteristic absorption bands of very strong intensities due to C=O (1691 cm^−1^) and COO- symmetric and asymmetric stretching (1577 and 1452 cm^−1^, respectively) as well as the absorption band at 3322 cm^−1^ due to N-H stretching characteristic of the HD2 polymorphic form. (Figure 1E). The spectrum also showed distinct absorption bands at 1506 cm^−1^ corresponding to C=C stretching vibrations in the aromatic ring and at 1282, 1199 and 1159 cm^−1^, due to C-N stretching in the secondary amines. Further, O-H stretching were visible in the region between 3000 and 2600 cm^−1^, and out of plane dimeric bending vibrations at 937 cm^−1^, while C-Cl stretching vibrations were evident at 765 and 740 cm^−1^. The ATR spectrum of the lyophilized NS T80 showed the characteristic bands of DCF acid and some typical bands of the stabilizer used for the formulation such as the C=O stretching (1733 cm^−1^) and the broad peak at 1100 cm^−1^. Moreover, the presence of the NH stretching peak at 3322 cm^−1^ typical of the of HD2 form in the NS T80 spectrum suggested that the polymorphic form of the original DCF is retained.

The DCF acid XRPD profile showed many high intensity signals that are characteristic of a crystalline structure (Figure 1D). From the comparison between NS T80 and DCF acid diffractograms it can be seen that nano sizing did not lead to change in crystalline form, although a slight decrease in intensity of peaks could be observed, confirming ATR results.

An important parameter for the nanosuspensions characterization is the saturation solubility [25]. The solubility of DCF as coarse suspensions or nanosuspensions was determined at 37 °C in distilled water (Table 2). As expected, the size reduction to nanocrystals increases the DCF water solubility of about 4 times (NS P188) and 7 times (NS T80) compared to coarse suspension prepared with the same stabilizer. DCF solubility is influenced not only by crystal size reduction but also by the surfactant employed. Tween 80 promotes the drug dissolution to a larger extent than Poloxamer 188, both when DCF is formulated as a coarse suspension or nanosuspension. Interestingly, the solubility of DCF as a P188-stabilized coarse suspension is not significantly higher than the solubility of the raw, unformulated material, previously reported (~20 µg/mL) [9].

Due to the higher solubility showed by NS T80, this formulation was employed for the full panel of skin penetration and permeation experiments in combination with the microneedle rollers, as well as for the electron microscopy analyses. Conversely, NS P188 were employed as a control in a smaller number of conditions.

A photograph of a microneedle roller used in this work and SEM images of the microneedles at different magnifications are reported in Figure 2A. The stainless-steel needles protrude orthogonally from the cylindrical surface of the roller with a length in accordance to what is declared by the manufacturer (0.2, 0.5 or 1.5 mm). The choice of a microneedle roller over a dissolvable/coated microneedle patch was based on the following considerations. First, stainless-steel needles are more resistant than polymeric needles, which structure may be affected by loading nanocrystals within their matrix. Second, coated microneedles are not suitable when a large amount of drug needs to be administered (e.g., in the case of DCF). Third, the use of microneedle rollers can be followed by the application of potentially any drug, making them much more versatile than dissolvable or coated microneedles (which require a dedicated manufacturing for any drug to be administered). Figure 2B allows a macroscopic visualization of the micropores formed on the skin upon microneedling with rollers having different needle lengths. In this experiment, pig skin specimens were treated with a nile red nanosuspension after the first, second, third and fourth applications of the roller in the directions described in the figure (left panel). Nile red was chosen because it can be formulated as nanosuspensions, and it can be easily visualized due to its intense red color [21]. Thus, using this simple setup, it was possible to have a preliminary, qualitative information about the extent of nanocrystals accumulation in the skin pores created by the microneedle rollers. In general, the micropores are clearly visible regardless of the needle length employed, and they increase in number at each passage of the roller. The intense red color of nile red nanocrystals is not visible on the intact skin specimens (Untreated, top row) nor in the spaces between each pore, suggesting that the action of the roller on the stratum corneum is limited to the formation of a well-ordered array of pores. In particular, using 0.5- and 1.5-mm microneedles (Figure 2B, second and third columns) the symmetric alignment of micropores is clearly visible and the generated pattern on the skin surface reflects the microneedles position on the roller. Moreover, the more intense red color suggests that longer needles produce wider/deeper pores that can accommodate larger amounts of nile red nanocrystals.

**Figure 3:**
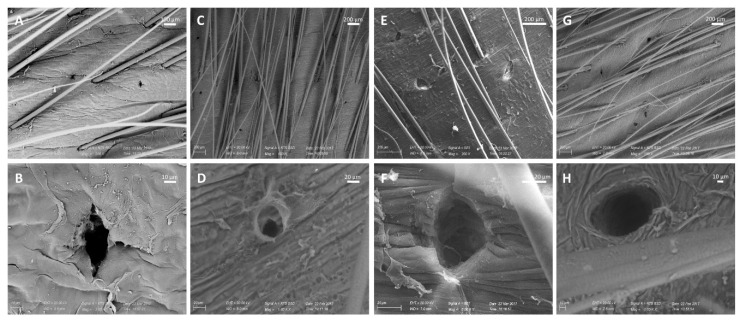
SEM visualization of skin pores after microneedle rolling in four directions with the DermaQ^®^ with needle lengths of 0.5 mm (**A**–**D**) or 1.5 mm (**E**–**H**). Images were acquired in HV mode ((**A**,**E**) 200× magnification and (**B**,**F**) 2000× magnification) or LV mode ((**C**,**G**) 100× magnification and (**D**,**H**) 1000× magnification).

Scanning electron microscopy (SEM) was used to observe more in detail the morphology of the pores created on the skin by two different types of DermaQ rollers (0.5- and 1.5-mm needle lengths). The samples were observed with two different operating modalities: high vacuum (HV), which requires a preparation of the biological sample (fixation, dehydration and coating) but allows for better image quality, and variable pressure (VP), which does not need any sample manipulation but leads to a loss in image resolution. In our opinion, the data provided by the two SEM mode are complementary and allow to rule out possible alterations to the skin structures due to the sample preparation. Importantly, images acquired in VP mode are more representative of the hydrated state of the skin that could be found in vivo and in vivo-simulated environments (e.g., when mounted on Franz cells) [26]. SEM confirms the formation of pores in the stratum corneum, with no damages in the surrounding area. Images at lower magnifications (Figure 3A,C,E,G) shows that the micro ducts are symmetrically aligned on the skin and have a rounded or slightly oblong shape. Higher magnifications (Figure 3B,D,F,H) allow to observe the pores in more detail and appreciate the different pore diameters depending on the microneedles used. In fact, rollers equipped with 0.5 mm microneedles produced pores of approximately 33 µm in diameter, while pores formed by 1.5-mm-long needles are about three times wider.

**Figure 4:**
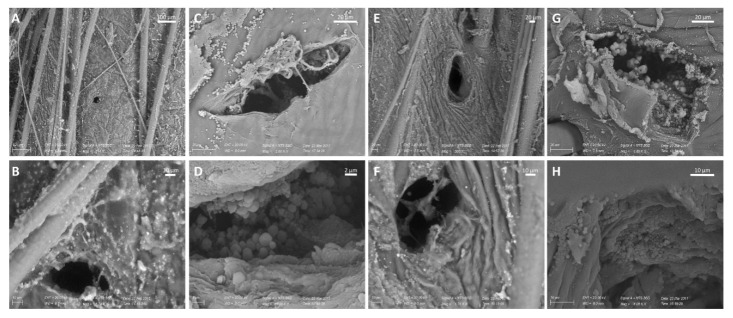
SEM visualization of DCF nanosuspensions (NST80) on the skin after microneedle rolling in four directions with the DermaQ^®^ with needle lengths of 0.5 mm (**A**–**D**) or 1.5 mm (**E**–**H**). Images were acquired in LV mode (**A**,**B**,**E**,**F**) or HV mode (**C**,**D**,**G**,**H**).

SEM was also used to visualize the skin samples treated with the combination of microneedle rollers and DCF nanosuspensions (Roll and Patch method). Figure 4 shows different magnifications and perspectives of the skin pierced with 0.5- and 1.5-mm microneedles and treated with nanosuspensions. The nanocrystals can be found on the skin surface and are clearly visible inside the channels created by both types of microneedles. Images acquired in HV mode allow for a clearer visualization of the round-shaped nanocrystals around and inside the micropores. Conversely, images acquired in VP mode only show bright spots on the skin surface, which can be identified as drug nanocrystals since the untreated skin does not show similar structures (Figure 3). The large number of nanocrystals deposited deeply within the pores suggests that the pre-treatment with microneedles could increase the dermal accumulation and/or transdermal penetration of DCF.

**Figure 5:**
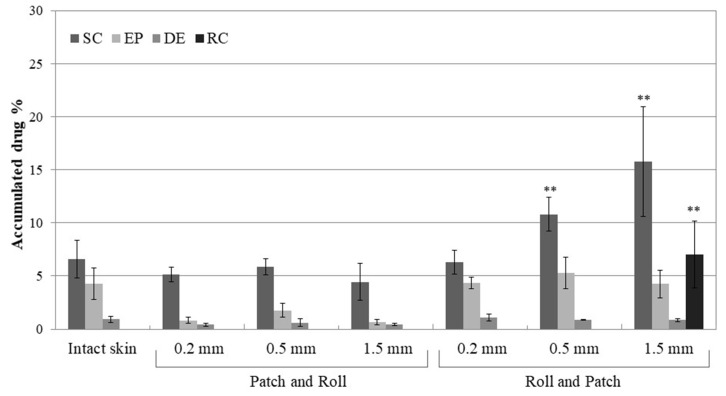
Cumulative amount of DCF retained into and permeated through the skin layers after 8 h treatment with nanosuspension NST80 and DermaQ^®^. The amount is expressed as the percentage of the dose applied on the skin. Patch and Roll: nanosuspension applied before microneedling. Roll and Patch: nanosuspension applied after microneedling. SC, stratum corneum; EP, epidermis; DE, dermis; RC, receptor compartment. ** *p* < 0.01 vs. Intact skin.

To investigate how the micropores influence the DCF distribution within the skin layers and below, we performed in vitro penetration and permeation studies using piglet skin and Franz diffusion cells. Newborn pig skin specimens mounted on Franz cells are a well-known in vitro system, widely accepted as a relevant model for the analysis of skin accumulation and permeation [27]. In the context of microneedle experiments, results obtained from this model should be analyzed keeping in mind that the skin is devoid of the healing processes occurring in vivo. In this experiment, microneedles with different needle lengths were used to pierce the skin before (Roll and Patch) or after the application of DCF nanosuspension (Patch and Roll). At the end of the experiment (8 h) the three skin layers (stratum corneum, epidermis, dermis) and the receptor compartment (simulating the sub-cutaneous tissue) were separated and analyzed for drug content. Figure 5 shows the percentage of DCF accumulated in the different skin layers upon treatment with NS T80 alone (control) or in combination with microneedling.

When NS T80 are applied on the intact skin, the drug is accumulated in the most superficial layers of the skin, with less than 1% recovered in the dermis and no transdermal penetration. Skin pre-treatment with microneedles (Roll and Patch) produces a significant increase of DCF in the stratum corneum, but only when 0.5- or 1.5-mm-long needles are employed. Pores obtained with shorter needles (0.2 mm) are not only less deep but also less wide than the ones obtained with 0.5- and 1.5-mm microneedles. Accessing such small, lipophilic aperture in the stratum corneum might be difficult for the nanosuspensions (that are water-based), which therefore accumulated similarly into the intact skin. Conversely, the use of longer needles is linked to higher amounts of DCF in the stratum corneum, probably due to the wider pores created on the skin (observed in Figure 3).

Wider pores can contain more material, especially in the top sections, closer to the skin surface and thus more accessible to the nanosuspension. The nanocrystals stuck on the walls of the pore tops can be slowly dissolved, releasing DCF that can diffuse laterally in the surrounding stratum corneum. In support of this view, the amount of DCF recovered in the epidermis and dermis was not significantly different when the skin was pre-treated by microneedles. Interestingly, the permeation of the drug across the skin barrier can only be achieved when the longest needles (1.5 mm) are employed.

When NS T80 is applied before the microneedling (Figure 5, Patch and Roll), a slight reduction of the total drug accumulated in the skin can be observed. Surprisingly, no differences arise from the use of rollers with different needle lengths. Reasons for this behavior might be found in the adhesive properties of the nanocrystals: The interactions quickly established with the skin surface prevent them from falling into the pores formed upon microneedling. Moreover, rolling in the four directions might remove part of the formulation from the skin surface, this being responsible for the reduced accumulation.

**Figure 6:**
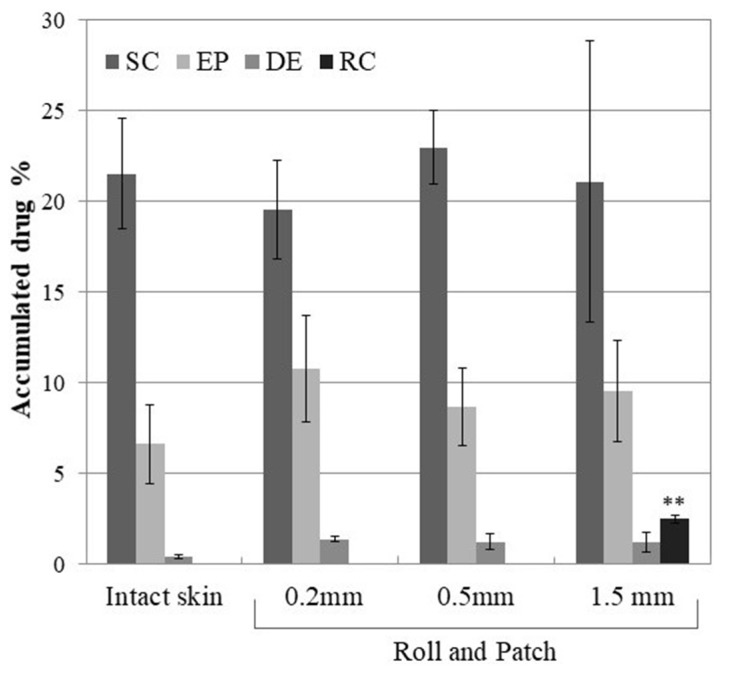
Cumulative amount of DCF retained into and permeated through the skin layers after 8 h treatment with nanosuspension NSP188 and DermaQ^®^. The amount is expressed as the percentage of the dose applied on the skin. Roll and Patch: nanosuspension applied after microneedling. SC, stratum corneum; EP, epidermis; DE, dermis; and RC, receptor compartment. ** *p* < 0.01 vs. Intact skin.

Lastly, the effect of microneedling (Roll and Patch) on the skin accumulation of DCF was investigated employing NS P188. NS P188 is a DCF nanosuspension stabilized by Poloxamer 188, previously developed in our lab and successfully employed to reduce skin inflammation in vivo [10]. As previously reported, the application of NS P188 on intact skin resulted in a marked accumulation in the top skin layers (Figure 6) [11].

When applied on the intact skin, this formulation outperformed the novel NS T80, showing a 3-fold higher amount of DCF in the stratum corneum. This behavior—in contrast with solubility data—suggests an ability of Poloxamer 188 to promote a stronger interaction between the stratum corneum and the nanocrystals. Disappointingly, the action of the microneedles rollers does not further improve the DCF accumulation, regardless of the needle lengths. This lack of efficacy might be linked to the cited P188-mediated adhesion of nanocrystals to the stratum corneum that induce the saturation of this tissue even without the use of microneedles. Similarly to the NS T80, DCF can be detected in the receptor compartment only when 1.5-mm microneedles are rolled on the skin. Given that the epidermal and dermal DCF content are not influenced by microneedling, it is reasonable to state that the 1.5-mm needles create a channel through the skin specimen, large enough to allow a small amount of nanocrystals to fall through it directly into the receptor compartment. This hypothesis is supported by the time-lapse analysis of DCF content in the receptor compartment, which shows that the majority of DCF can be detected right after the application of NS T80 on the skin (Figure S2).

## 4. Conclusions

Increasing the amount of locally delivered and retained DCF can improve its safety and efficacy against skin and musculo-skeletal inflammations. To this aim, here we combined a nanotechnological approach with a device to physically disrupt the skin barrier. DCF nanosuspensions stabilized with Tween 80 (NS T80) were prepared and characterized for the first time, and their efficacy in terms of skin penetration and accumulation was compared to a previously developed formulation (NS P188). The action of microneedle rollers with different needle lengths on porcine skin was evaluated by macro- and microscopic imaging. The two technologies combined increase the amount of DCF accumulated in the stratum corneum due to the formation of nanocrystal depots within skin pores but only when the application of NS T80 follows the microneedling with 0.5- or 1.5-mm-long needles. Importantly, the accumulation enhancement is highly dependent on both the microneedles and the nanosuspensions properties, and it might not be appreciable when a nanoformulation has good intrinsic skin accumulation (e.g., NS P188).

## Figures and Tables

**Table 1:** Composition, average diameter, polydispersity index (PI) and Zeta potential of Diclofenac acid (DCF) nanosuspension after milling. Each value is the mean ± standard deviation of at least six experimental determinations. Statistical analysis has been performed comparing the mean diameter, PI or Zeta potential value of NST80 with respect to that of NSP188. * = statistically different values (*p* ≤ 0.05), ns indicates not significant data (*p* > 0.05).

Formulations	Component	Stabilizer	Properties		
			Particle Size (nm)	PI	Zeta Potential (mV)
NS T80	DCF 1% (*w*/*w*)	Tween 80 0.5% (*w*/*w*)	264 ± 6.8	0.17 ± 0.04	−51 ± 0.6
NS P188	DCF 1% (*w*/*w*)	Poloxamer 188 0.5% (*w*/*w*)	279 ± 7.7 ^ns^	0.17 ± 0.01 ^ns^	−35 ± 1.3 *

**Table 2:** Water solubility of DCF coarse suspensions (CS P188, CS T80) and nanosuspensions (NS P188 and NS T80) at 37 °C.

Formulation	Solubility (µg/mL ± SD)
CS P188	30.40 ± 1.93
CS T80	61.33 ± 7.33
NS P188	72.97 ± 6.81
NS T80	148.90 ± 7.96

## Supplementary Materials

The following are available online at https://www.mdpi.com/1999-4923/12/12/1140/s1, Figure S1: DSC thermograms of bulk DCF and DCF nanocrystals (NS T80); Figure S2: Amount of DCF detected in the receptor compartment at different time points after the treatment with microneedle rollers (needle length 1.5 mm) and application of NS T80 (Roll and patch method). The amount is expressed as the percentage of the dose applied on the skin.
