# Supplementary material for: Nanosuspensions and Microneedles Roller as a Combined Approach to Enhance Diclofenac Topical Bioavailability
Source: Pharmaceutics. 2020 Nov 25;12(12):1140. doi: 10.3390/pharmaceutics12121140 (PMC7760567; doi:10.3390/pharmaceutics12121140)
Supplement: Supplementary file 1 [file pharmaceutics-12-01140-s001.pdf]

pharmaceutics

MDPI

# Supplementary Materials: Nanosuspensions and Microneedles Roller as A Combined Approach to Enhance Diclofenac Topical Bioavailability

Rosa Pireddu, Michele Schlich, Salvatore Marceddu, Donatella Valenti, Elena Pini, Anna Maria Fadda, Francesco Lai* and Chiara Sinico

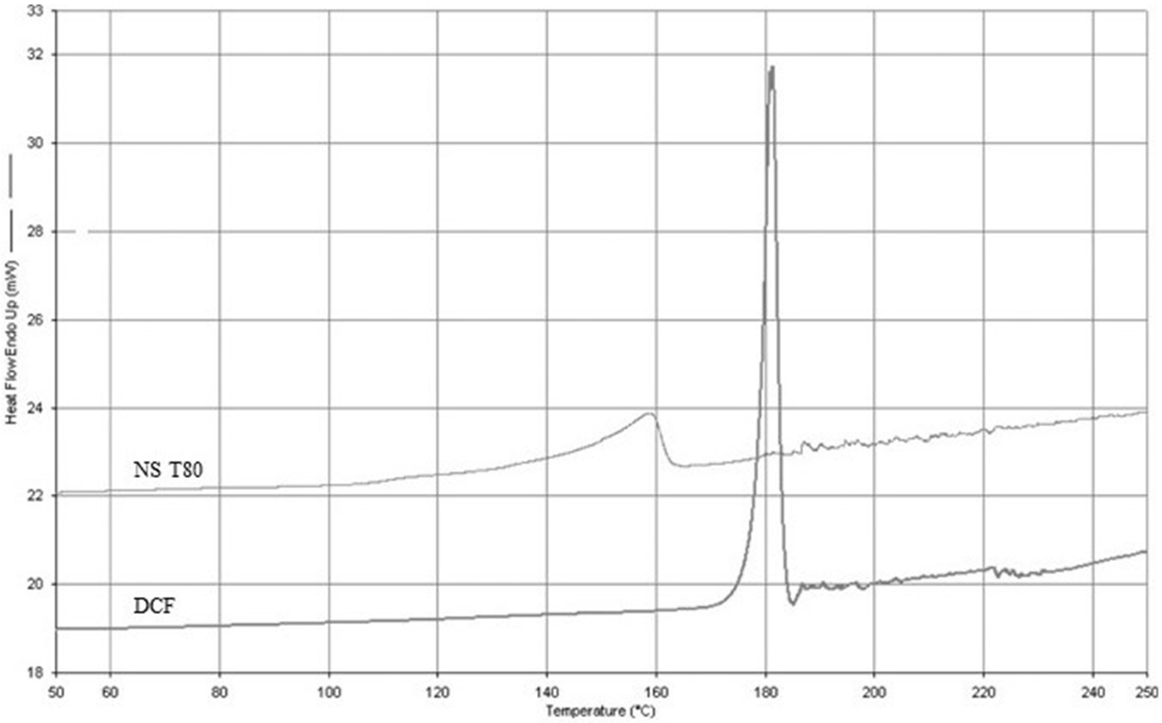


**Figure S1.** DSC thermograms of bulk DCF and DCF nanocrystals (NS T80).

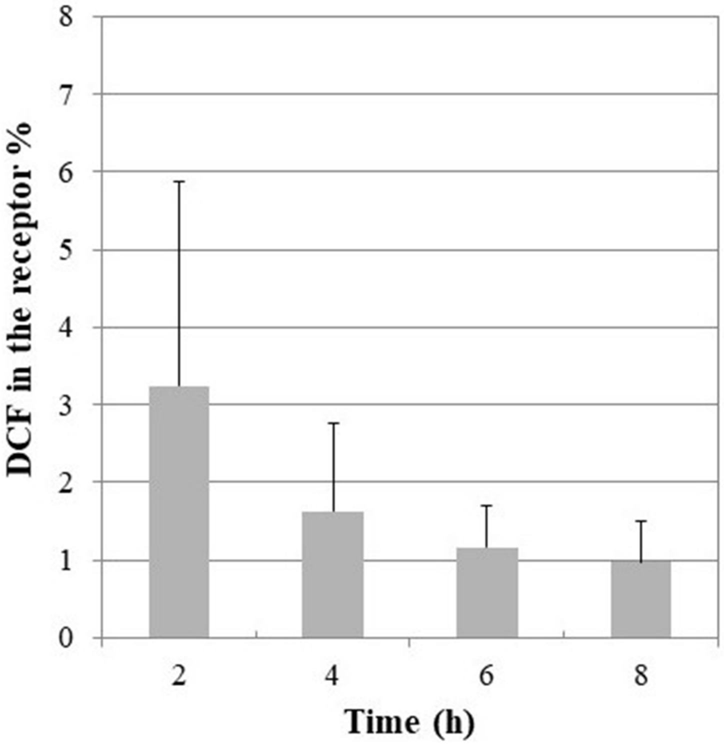


**Figure S2.** Amount of DCF detected in the receptor compartment at different time points after the treatment with microneedle rollers (needle length 1.5 mm) and application of NS T80 (Roll and patch method). The amount is expressed as the percentage of the dose applied on the skin.
